# Toward an ecoregion scale evaluation of eDNA metabarcoding primers: A case study for the freshwater fish biodiversity of the Murray–Darling Basin (Australia)

**DOI:** 10.1002/ece3.4387

**Published:** 2018-08-05

**Authors:** Jonas Bylemans, Dianne M. Gleeson, Christopher M. Hardy, Elise Furlan

**Affiliations:** ^1^ Institute for Applied Ecology University of Canberra Canberra ACT Australia; ^2^ Invasive Animals Cooperative Research Centre University of Canberra Canberra ACT Australia; ^3^ CSIRO Land and Water Canberra ACT Australia

**Keywords:** environmental DNA, high‐throughput sequencing, in silico, metabarcoding, primers

## Abstract

High‐throughput sequencing of environmental DNA (i.e., eDNA metabarcoding) has become an increasingly popular method for monitoring aquatic biodiversity. At present, such analyses require target‐specific primers to amplify DNA barcodes from co‐occurring species, and this initial amplification can introduce biases. Understanding the performance of different primers is thus recommended prior to undertaking any metabarcoding initiative. While multiple software programs are available to evaluate metabarcoding primers, all programs have their own strengths and weaknesses. Therefore, a robust in silico workflow for the evaluation of metabarcoding primers will benefit from the use of multiple programs. Furthermore, geographic differences in species biodiversity are likely to influence the performance of metabarcoding primers and further complicate the evaluation process. Here, an in silico workflow is presented that can be used to evaluate the performance of metabarcoding primers on an ecoregion scale. This workflow was used to evaluate the performance of published and newly developed eDNA metabarcoding primers for the freshwater fish biodiversity of the Murray–Darling Basin (Australia). To validate the in silico workflow, a subset of the primers, including one newly designed primer pair, were used in metabarcoding analyses of an artificial DNA community and eDNA samples. The results show that the in silico workflow allows for a robust evaluation of metabarcoding primers and can reveal important trade‐offs that need to be considered when selecting the most suitable primer. Additionally, a new primer pair was described and validated that allows for more robust taxonomic assignments and is less influenced by primer biases compared to commonly used fish metabarcoding primers.

## 1 INTRODUCTION

Obtaining accurate biodiversity estimates is critical for effective management of our natural resources (Dudgeon et al., 2006; Maxwell & Jennings, 2005). PCR amplification of small barcode sequences from environmental DNA (eDNA) combined with high‐throughput sequencing (HTS) technologies, commonly referred to as eDNA metabarcoding, has become an increasingly popular tool for monitoring biodiversity (Bohmann et al., 2014; Cristescu, 2014; Taberlet, Coissac, Pompanon, Brochmann, & Willerslev, 2012). However, primers used in the initial amplification of barcode sequences can introduce significant biases (Clarke, Beard, Swadling, & Deagle, 2017; Elbrecht & Leese, 2015; Tremblay et al., 2015). Even though careful primer selection is widely recognized to be a crucial step prior to undertaking metabarcoding initiatives, selecting the most appropriate primers can be a complex task.

Ideally, primers for eDNA metabarcoding should: (a) amplify a short DNA fragment (i.e., typically <150–200 bp long) to maximize the recovery of DNA from environmental samples, (b) amplify a barcode with sufficient taxonomic resolution to allow for robust species assignments, (c) be specific to the taxonomic group of interest to avoid amplification and subsequent sequencing of nontarget taxa, and (d) amplify DNA from all species of interest with equal efficiency to minimize primer biases (Clarke et al., 2017; Coissac, Riaz, & Puillandre, 2012; Elbrecht & Leese, 2017). While commonly used barcoding primers allow for robust species identification, they are often unsuitable for eDNA metabarcoding applications. For example, the commonly used cytochrome *c* oxidase subunit I (COI) gene lacks highly conserved regions needed for robust metabarcoding primer design (Deagle, Jarman, Coissac, Pompanon, & Taberlet, 2014). While incorporating a high degree of base degeneracy can improve the performance of COI primers (Elbrecht & Leese, 2017), mitochondrial ribosomal RNA (rRNA) gene regions are increasingly being used to minimize primer‐template mismatches, and the amplified barcodes have a taxonomic resolution similar to standard COI barcodes (Kocher et al., 2017; Riaz et al., 2011; Valentini, Pompanon, & Taberlet, 2009). Given that there are no truly “universal” metabarcoding primers, selecting the most suitable primers will always require balancing the trade‐offs that exist between the four criteria mentioned previously (Valentini et al., 2016). First, a positive relationship exists between the length of the internally amplified barcode and its taxonomic resolution power (Coissac et al., 2012; Meusnier et al., 2008) but the ability to recover DNA from environmental samples can be negatively impacted by the size of the DNA fragments (Deagle, Eveson, & Jarman, 2006; Jo et al., 2017). However, a number of recent studies have shown that this may be less problematic for eDNA derived from water samples (Bylemans, Furlan, Gleeson, Hardy, & Duncan, 2018; Deiner et al., 2017; Piggott, 2016). Second, reducing primer‐template mismatches can minimize biases arising from the PCR amplification but may inadvertently decrease the specificity of the primers to the taxonomic group of interest as these primers are more likely to bind to highly conserved regions (Pinol, Mir, Gomez‐Polo, & Agusti, 2015). In addition to these trade‐offs, species biodiversity varies extensively between geographic regions (Abell et al., 2008; Olson et al., 2001; Spalding et al., 2007) which further complicates the selection process as the performance of metabarcoding primers will vary depending on the species composition at the sampling location. In recent years, a number of software programs have been developed for the in silico evaluation of metabarcoding primers (Boyer et al., 2012; Cannon et al., 2016; Elbrecht & Leese, 2016; Ficetola et al., 2010; Foster, Sharpton, & Grünwald, 2017; Riaz et al., 2011). However, most studies to date have only evaluated primers using a single program, and the performance of primer performance has not been evaluated at a regional scale.

The aim of this study was to provide an in silico workflow for the evaluation of metabarcoding primers at an ecoregion scale. The overall workflow utilizes customized genetic databases and multiple available software programs. We subsequently used our proposed workflow to evaluate the performance of existing and newly developed metabarcoding primers for the freshwater fish biodiversity of the Murray–Darling Basin (MDB; Australia). The MDB is Australia's largest river catchment covering approximately 14% of its area and spanning 5 states (Lintermans, 2007). A total of 62 freshwater fish species currently occur within the MDB, and approximately 32% of the native fish species are endemic to the MDB (Adams, Raadik, Burridge, & Georges, 2014; Lintermans, 2007; Raadik, 2014; Unmack, 2013). A subset of all primer pairs were used in metabarcoding analyses of an artificial DNA community and eDNA samples to evaluate the performance of the in silico workflow and validate one of the newly designed primer pair.

## 2 MATERIALS AND METHODS

### 2.1 Workflow for in silico primer evaluation

#### 2.1.1 Literature review and primer development

A literature search was conducted for available metabarcoding primers. The search was restricted to primers specifically designed for fish species and eDNA applications. Furthermore, new metabarcoding primers were designed specifically for freshwater *Actinopterygii* species occurring in the MDB. Primer design focussed on the mitochondrial 12S ribosomal RNA gene as it has been shown previously that relative short DNA fragments of this gene are able to uniquely identify most species occurring in the MDB (Hardy et al., 2011). Tissue samples and/or DNA extracts were obtained for all *Actinopterygii* species, and the 12S ribosomal RNA gene was PCR amplified and Sanger sequenced (Appendix S1). Primers were designed to bind to highly conserved regions while flanking highly variable regions. No restrictions were set on amplicon length as previous studies have shown that relatively large mitochondrial DNA fragments can be successfully amplified from aquatic eDNA (Deiner et al., 2017; Piggott, 2016; Sigsgaard et al., 2016). Primers were designed with a 30%–80% GC content and a melting temperature between 50 and 60°C. The maximum allowed difference in melting temperature between the forward and reverse primer was 1.5°C. If primer‐binding regions contained C/T or A/G variable sites, primers contained a G or C, respectively, to take into consideration the atypical base pairing in T/G bonds (Miya et al., 2015). Newly developed primers were evaluated in silico for undesirable primer interactions using the Beacon Designer™ Free Edition software (PREMIER Biosoft, Palo Alto, CA, USA), and those primer pairs forming highly stable secondary structures were excluded from further analyses.

#### 2.1.2 Initial screening of metabarcoding primers

An initial screening of all published and newly developed metabarcoding primers was performed to reduce the number of primer pairs for further analyses. PCR amplification was simulated in silico for each primer pair using publicly available genetic data repositories. Multiple software programs are available to query primers against online databases (Cannon et al., 2016; Ficetola et al., 2010; Foster et al., 2017). Here, the R package PrimerTree was used because of its ease of use and speed of execution (Cannon et al., 2016). For each primer pair, a random subset of 1,000 amplifiable sequences was retrieved from the NCBI nucleotide database using the search_primer_pair function, and summary statistics were calculated based on the obtained PrimerTree objects. First, the taxonomic resolution of the barcoding regions was evaluated by calculating the average number of bp differences between species with the calc_rank_dist_ave function. The taxonomic resolution power of the amplified barcodes was expressed as the average number of bp differences per 100 bases to allow for comparisons between primers amplifying barcoding regions of different lengths. Second, the percentage of unique sequence records belonging to *Actinopterygii* species was determined and used to assess the specificity of primer pairs. At last, the taxonomic coverage of the primers was evaluated by determining the number of Actinopterygii orders for which sequences records were obtained. The calculated statistics were subsequently used to select the best performing primers for further analyses. Primers were considered to pass the initial screening when the amplified barcodes contained on average more than 5 bp differences per 100 bases, more than 90% of all species for which sequences were recovered belonged to the Actinopterygii class, and sequences were amplified in silico for more than 30 Actinopterygii orders.

#### 2.1.3 Evaluate primer specificity and primer bias

The R package PrimerMiner (Elbrecht & Leese, 2016) was used to simultaneously evaluate the specificity of the metabarcoding primers and to assess the impact of primer biases on the amplification efficiency. While other programs such as ecoPCR can be used to evaluate the specificity of metabarcoding primers (Ficetola et al., 2010), PrimerMiner is currently the only packages, which evaluates amplification success taking into consideration the adjacency, position, and type of bp mismatches between primer and templates (Elbrecht & Leese, 2016).

First, databases were constructed for all gene regions targeted by those primers that passed the initial screening. Genetic databases were constructed by batch downloading 12S and 16S sequence records from the NCBI database (accessed October 2017) using PrimerMiner v.0.15. For each gene region, sequences were downloaded for all major vertebrate classes (i.e., Actinopterygii, Chondrichthyes, Amphibia, Reptilia, Aves, and Mammalia). A customized taxonomic table was used to exclusively downloaded sequences for those taxonomic families with occurrence records within the Darling River drainage (Atlas of Living Australia; Appendix S1, Table S2). The configuration file for downloading sequences was modified to download 12S (Marker = c(“12S”, “s‐rRNA”, “rrnS”, “12S ribosomal RNA”) and 16S (Marker = c(“16S”, “l‐rRNA”, “rrnL”, “16S ribosomal RNA”) sequences from the NCBI database (download_bold = F) and cluster sequence records into operational taxonomic units (OTU) using a 3% sequence similarity.

For each gene, all sequence records extracted from whole mitochondrial genomes were imported into Geneious v8.1.8 and a mafft alignment was constructed (Kearse et al., 2012). Primer annotations were added to the consensus sequence and gaps in the primer‐binding regions were manually removed before extracting the 50% consensus sequence. OTU sequence records for each gene region and vertebrate class were mapped against the 50% consensus sequence resulting in 12 OTU alignments (i.e., 2 gene regions × 6 vertebrate classes). The de novo generated 12S sequences from all *Actinopterygii* species were combined with the Actinopterygii OTU sequences prior to mapping sequences against the 50% consensus sequence. A custom R script was used to clean the OTU alignments by (a) removing all positions for which the alignment created gaps in the consensus sequence, (b) removing the consensus sequence, and (c) deleting sequences with more than 2% ambiguous bases and coverage below 30% of the total length of the alignment (Appendix S2). The evaluate_primer and primer_threshold functions in PrimerMiner were subsequently used to evaluate the amplification success of the primer pairs for each vertebrate class. Threshold values used to evaluate amplification success ranged from 10 to 300 with a constant interval of 10 with higher threshold values allow for more primer‐template mismatches.

#### 2.1.4 Compare the taxonomic resolution

Programs such as ecoPCR, BarcodingR, and SPIDER can provide metrics to evaluate the taxonomic resolution of barcoding regions (Boyer et al., 2012; Ficetola et al., 2010; Zhang, Hao, Yang, & Shi, 2017). The latter two are R packages which can be easily integrated into the Rscript used for the initial screening of the primers and to evaluate primer specificity and primer bias (Appendix S2). However, here we used the ecoPCR scripts within OBITools to evaluate the taxonomic resolution power of the internally amplified barcodes as the OBITools scripts will also be used for the bioinformatics analyses of the eDNA metabarcoding data (see Section 2.2).

All standard vertebrate sequences from the EMBL data repository (release 132) were downloaded prior to simulating an in silico PCR with the ecoPCR script for each primer pair (allowing for a maximum of 3 bp mismatches for each primer). All sequences belonging to Actinopterygii families occurring in the MDB were subsequently extracted (Appendix S1, Table S2). Additionally, an in silico PCR was performed using the de novo generated 12S sequences for those primers targeting the 12S gene. The in silico amplified barcodes obtained from the EMBL database and the custom 12S database were combined for each primer pair, and the ecotaxspecificity script was used to evaluate the taxonomic resolution of the barcodes at the genus and species level with a low (i.e., 2 bp differences) and a high (i.e., 5 bp differences) threshold for barcode similarity.

### 2.2 Metabarcoding analyses

One of the newly developed primer pairs (i.e., AcMDB07) performed well based on in silico analyses (see Section 3). To validate this novel primer pair and simultaneously evaluate the performance of the in silico analyses, the three primer pairs targeting the 12S gene region (i.e., MiFish‐U, Teleo, and AcMDB07) were used in metabarcoding analyses of artificial community (AC) sample and eDNA samples collected from two locations within the MDB (Miya et al., 2015; Valentini et al., 2016).

#### 2.2.1 Sample description

An AC was constructed using PCR amplicons of the entire 12S gene region from ten fish species to evaluate the impact of primer‐template mismatches for each primer pair. Species showing varying levels of primer‐template mismatches were selected based on the PrimerMiner penalty scores (Table 1). Amplicon concentrations were quantified using the Qubit dsDNA BR Assay Kit (Invitrogen) and converted to copy numbers per μL. All amplicons were diluted to 1 × 10^3^ copies per μL before combining equal volumes from each amplicon to form the AC.

**Table 1:** Species used to construct the artificial community (AC) and the PrimerMiner penalty scores for each species and primer pair. The MiFish‐U and Teleo primer pairs have previously been validated (Miya et al., 2015; Valentini et al., 2016). The AcMDB07 primer pair was designed in the current study

Taxonomic family	Species name	PrimerMiner penalty score		
		MiFish‐U	Teleo	AcMDB07
Anguillidae	*Anguilla australis*	6.20	84.90	18.45
Terapontidae	*Bidyanus bidyanus*	6.20	71.20	0.00
Cyprinidae	*Cyprinus carpio*	6.20	84.90	18.45
Gadopsidae	*Gadopsis marmoratus*	6.20	13.60	4.65
Galaxiidae	*Galaxias maculatus*	57.20	0.00	0.00
Eleotridae	*Mogurnda adspersa*	6.20	0.00	42.25
Plotosidae	*Neosilurus hyrtlii*	27.85	106.55	6.20
Salmonidae	*Oncorhynchus mykiss*	6.20	0.00	42.25
Plotosidae	*Porochilus rendahli*	27.85	106.55	6.20
Retropinnidae	*Retropinna semoni*	127.00	0.00	0.00

Environmental DNA samples collected from two sites within the MDB were used to compare the fish community data obtained from each primer pair. Water samples were collected from a single site within Blakney Creek (BC; 34°38′38.04″S and 149°2′11.796″E) and the Murrumbidgee River (MR; 35°19′8.554″S and 148°57′29.4998″E) during October and November 2016, respectively. Potential contaminant DNA was removed from sampling equipment using a 20% bleach solution and thoroughly rinsing with UV‐sterilized tap water. A total of 8 and 12 two liter water samples were collected from the BC and MR sites, respectively. One Blank Field Control (BFC) was included for each sampling site and consisted of a 2‐L sampling bottle filled with UV‐sterilized tap water which was opened on site, closed, and submerged in the water. All samples were stored on ice and transported to the University of Canberra for filtering. Filtering equipment was sterilized as described above and Negative Equipment Controls (NECs) were obtained by filtering 500 ml of UV‐sterilized tap water before filtering the field samples. A 1.2‐μm glass fiber filter (Sartorius, Göttingen, Germany) was used to capture eDNA within 12 hr after sample collection and filters were stored at −20°C. Environmental DNA was extracted using the PowerWater DNA Extraction Kit (MoBio Laboratories, Carlsbad, CA, USA) in the trace DNA laboratory at the University of Canberra. Negative controls (i.e., one BFC for each site, one NEC for the BC site, and two NECs for the MR site) were included in the batch DNA extractions to monitoring potential contamination. All eDNA extracts were subsequently stored at −20°C until further processing.

#### 2.2.2 PCR amplification and library preparation

Prior to constructing HTS libraries, negative controls were screened for the presence of fish eDNA. Real‐time PCRs were run in triplicate for each sample and primer pair combination to minimize the effects of PCR stochasticity. PCRs consisted of 0.20 μl of AmpliTaq Gold DNA Polymerase (5 U/μl; Applied Biosystems, Foster City, CA, USA), 2.50 μl of GeneAmp 10× Gold Buffer (Applied Biosystems), 2.00 μl of MgCl_2_ (25 mmol/L; Applied Biosystems), 0.65 μl of GeneAmp dNTP Blend (10 mmol/L; Applied Biosystems), 0.20 μl UltraPure BSA (50 mg/ml; Invitrogen), 0.60 SYBR Green I Nucleic Acid Gel Stain (5X; Invitrogen), 1.00 μl of each primer (10 μmol/L), and 4.00 μl of template DNA and DEPC‐treated water (Invitrogen) to a final volume of 25 μl. All PCRs were run using a Bio‐Rad CFX96 Real‐Time PCR System (Bio‐Rad Laboratories, Hercules, USA). Annealing temperatures (*T*_A_) used for each primer pair were determined experimentally by running a gradient real‐time PCR for each primer pair with template DNA consisting of both fish genomic DNA and eDNA. Optimal annealing temperatures were selected based on the *C*_t_ values and the shape of the melt curves. Annealing temperatures used were: 61.5°C for the MiFish‐U primers, 55°C for the Teleo primers, and 53°C for the AcMDB07 primers. PCR thermal cycling conditions consisting of an initial activation step of 5 min at 95°C; 45 3‐step cycles of 30 s at 95°C, 30 s at T_A,_ and 1 min at 72°C; and a final extension of 10 min at 72°C and a melting curve with a stepwise increase of 0.1°C/5 s from 60 to 95°C. When positive amplification was observed for a negative control sample, these samples were included in the library preparation for HTS.

The construction of HTS libraries for each sample and primer pair was undertaken using a one‐step amplification. A single PCR step with fusion tagged primers (FTP) was used to amplify the barcoding sequence and add technical sequences required for HTS. Forward FTP consisted of the P5 sequencing adaptor, a custom forward sequencing primer, a 7 bp Multiplex Identification (MID‐) tag, and the forward fish‐specific primer. Reverse FTP contained the P7 sequencing adaptor, a custom reverse sequencing primer, a 7 bp MID‐tag, and the fish‐specific reverse primer. MID‐tags were generated using edittag scripts, and unique combinations of forward and reverse MID‐tags were used to label PCR amplicons (Faircloth & Glenn, 2012). Triplicate PCRs were run for each unique primer combination using the reaction conditions and thermal cycling profile described previously. Three uniquely labeled libraries were constructed for the AC sample for each primer pair (i.e., three unique FTP combinations with three PCR replicates for each combination). For all other samples (i.e., eDNA and negative control samples), a single uniquely labeled library was constructed. Based on the average Ct value of each sample, amplicon libraries of 9–12 samples were pooled using equal volumes of each PCR replicate. Even if no amplification was observed for the negative control samples low amplicon concentrations may still be present. Thus, aliquots of negative PCRs were included in the pooling step and were combined with those samples having the highest average Ct values. Excess FTP and primer dimer was removed using Agencourt AMPure XP Beads (Beckman Coulter, Brea, CA, USA) in a 1.2 volume ratio relative to the amplicon pool. The NanoDrop^®^ ND‐1000 spectrophotometer (Thermo Fisher Scientific, Waltham, MA, USA) was used to quantify amplicon concentration in each pool prior to combining them into a single super pool. The super pool was constructed by combining approximately equal amplicon copy numbers from each initial pool (i.e., taking into account the number of samples combined during the first pooling step and the amplicon size). A total of 75 uniquely labeled libraries from this study (i.e., 69 and 6 libraries originating from eDNA and negative control samples, respectively) and 168 libraries generated for a different project were included in the final super pool. A final cleanup step was conducted for the super pool as described previously. At last, all 243 libraries were sequenced using a paired‐end MiSeq run with the v3 2x300 bp sequencing kit at the Ramaciotti Centre for Genomics (University of New South Wales).

#### 2.2.3 Data analyses

Sequencing adaptors and sequencing primers were trimmed from the paired‐end reads using Trimmomatic v.0.36 (Bolger, Lohse, & Usadel, 2014). The obitools software package was used for subsequent filtering of the sequences following the general workflow as described by De Barba et al. (2014). The pairedend and ngsfilter scripts were used to assemble forward and reverse sequence reads and assign sequences to the corresponding samples, respectively. In addition to all FTP combination used to construct the HTS libraries, unused primer combinations were included during the sample assignments step. The obisplit script was then used to create separate files for each primer pair and sample. Unique sequences were clustered using the obiuniq script before removing short sequences (i.e., remove sequences below 150, 50, and 250 bp for the MiFish‐U, Teleo, and AcMDB07 primers, respectively) and sequences with low occurrences. For the AC data, only sequences with a single occurrence were removed, while for all other samples, sequences with an occurrence lower than 120 were removed. The 120 threshold was determined experimentally so that all sequences assigned to *Actinopterygii* species were removed from negative control samples (i.e., the highest occurrences were observed for unused combinations of FTP). PCR and sequencing errors were removed using the obiclean and obigrep script (i.e., remove all sequences identified as “internal” by the obiclean script). The sequences from each primer pair were combined into a single file, and unique sequences were clustered while retaining the individual sample information. The ecotag script was used to assign taxonomic information to the sequences using a reference database build using the standard vertebrate sequences from the EMBL data repository (release 132) and the custom 12S sequences of all *Actinopterygii* species in the MDB. Only custom 12S sequences were used for the Actinopterygii families occurring in the MDB to obtain more precise taxonomic assignments. At last, the change in sequence abundance throughout the bioinformatics filtering process was monitored on a per sample basis using the obistat script.

Further filtering and analyses of the metabarcoding data were achieved using the packages tidyverse, vegan, lme4, broom, and gridExtra in R version 3.4.1 (Appendix S3; Auguie, 2012; Bates, Maechler, Bolker, & Walker, 2014; Oksanen et al., 2007; R Development Core Team, 2010; Robinson, 2014; Wickham, 2016). For the AC data, some low abundant sequences were assignment to higher taxonomic ranks than the species level. Given that all species included in the AC are known, and all incorrectly assigned sequences had correctly assigned variants with a higher occurrence, these incorrect assignments were reassigned to the correct species. For the data obtained from the eDNA samples, the data were evaluated on a case‐by‐case basis. Ambiguous taxonomic assignments were modified/corrected taking into consideration the relative sequence abundance, the sequence information, the barcode resolution, and the a priori knowledge of the fish biodiversity at each sampling site. For example, all sequences assigned to *Galaxias* species were combined into a single genus level assignment, as the barcode resolution for all primer pairs is insufficient to resolve species within the closely related *Galaxias* complex. Additionally, sequences assigned to the *Nannoperca* genus obtained from the BC site for the Teleo and AcMDB07 primers were reassigned to *Nannoperca australis* as the only other *Nannoperca* species (*Nannoperca obscura*) does not occur in this river system (Lintermans, 2007). All sequences without a taxonomic assignment or with assignments to nonfish vertebrate species were clustered together and excluded from further analyses.

The data from the AC sample were used to evaluate the impact of primer‐template mismatches on the proportional read abundance (PRA). As the AC consisted of equal amplicon copies of 10 species, a PRA of 0.1 was expected for each species. However, primer‐template mismatches can result in unequal amplification efficiency and can skew the PRA data. Thus, the PRA is expected to be higher for species with a perfect match between the primers and the template DNA and will decrease with increasing mismatches (i.e., higher PrimerMiner penalty scores). When fitting a linear model to the PRA data as a function of the primer‐template mismatches (i.e., PrimerMiner penalty scores) a slope close to zero will thus indicate an equal amplification efficiency for all species, while more negative values are expected for primers with a biased amplification. The PRA data were logit‐transformed to achieve normality (Equation (1)) before fitting a linear mixed‐effect model for each primer pair. The logit‐transformed PRA was set as the response variable and the PrimerMiner penalty scores as fixed effects. PCR replicates, originating from the three different FTP combinations used for HTS library preparation, were included as random effects. Regression slope estimates were compared between the different primers to assess the impacts of primer‐template mismatches on the amplification efficiency.

(1) $$\text{logit}\left(\text{PRA}\right)=\log\left(\text{PRA}/(1-\text{PRA})\right)$$

The data obtained for the two field sampling sites were used to evaluate the number of species detected for each primer and assess community‐level differences between the different primers. The effect of sampling intensity and sequencing depth on the species richness detected at each site was evaluated using the community data with the absolute read abundances for each species. A custom R script (Appendix S3) was used to rarefy the community data to represent different levels of sequencing depth (i.e., 10,000; 30,000, and 60,000 reads per sample) while also taking into consideration the number of sequence reads discarded per sample during the bioinformatics filtering process. Low abundant detections (i.e., with a count below 120) were removed, and the community data were transformed to the presence/absence data. At last, species accumulation curves were constructed using the specaccum function within the R package vegan for each primer pair, sampling site, and sequencing depth combination. The community data were transformed to both the presence/absence data and proportional abundances to evaluate community‐level differences for the different metabarcoding primers. Analyses of variance were performed using the adonis function (R package vegan) for each sampling site and both data sets with the community matrix as the dependent variable and primers as independent variables. When primers had a significant effect on the community data, the simper function (R package vegan) was used to estimate the overall dissimilarity between the primer pairs and evaluate the average contribution of each species.

## 3 RESULTS

### 3.1 In silico primer evaluation

Eight fish‐specific primer pairs were retrieved from the available literature, and seven additional metabarcoding primers were designed specifically for fish species in the MDB. One of the newly designed primers was excluded from further in silico analyses as it formed highly stable secondary structures and is unlikely to be suitable. A total of 14 primers were used in the in silico analyses, and the details of these primer pairs are given in Table 2.

**Table 2:** Primer pairs used during the in silico analyses

Primer ID	Direction	Primer sequence (5′‐3′)	Amplicon
FishCB^a^	Forward	TCCTTTTGAGGCGCTACAGT	*ca*. 130 bp
	Reverse	GGAATGCGAAGAATCGTGTT	
16S1^b^	Forward	CGAGAAGACCCTWTGGAGCTTIAG	*ca*. 107 bp
	Reverse	GGTCGCCCCAACCRAAG	
Ac16s^c^	Forward	CCTTTTGCATCATGATTTAGC	*ca*. 375 bp
	Reverse	CAGGTGGCTGCTTTTAGGC	
16S2^d^	Forward	GACCCTATGGAGCTTTAGAC	*ca*. 233 bp
	Reverse	CGCTGTTATCCCTADRGTAACT	
16S‐Fish^e^	Forward	AGCGYAATCACTTGTCTYTTAA	*ca*. 233 bp
	Reverse	CRBGGTCGCCCCAACCRAA	
Ac12s^c^	Forward	ACTGGGATTAGATACCCCACTATG	*ca*. 429 bp
	Reverse	GAGAGTGACGGGCGGTGT	
MiFish‐U^f^	Forward	GTCGGTAAAACTCGTGCCAGC	*ca*. 219 bp
	Reverse	CATAGTGGGGTATCTAATCCCAGTTTG	
Teleo^g^	Forward	ACACCGCCCGTCACTCT	*ca*. 100 bp
	Reverse	CTTCCGGTACACTTACCATG	
AcMDB01	Forward	GGGAAGAAATGGGCTACA	*ca*. 227 bp
	Reverse	TACACTTACCATGTTACGACT	
AcMDB02	Forward	CAAACTGGGATTAGATACCCCACTATG	*ca*. 147 bp
	Reverse	GGTTCTAGGACAGGCTCCTCTAG	
AcMDB03	Forward	CAAACTGGGATTAGATACCCCACTATG	*ca*. 149 bp
	Reverse	CGGTTCTAGGACAGGCTCCTC	
AcMDB04	Forward	CAAACTGGGATTAGATACCCCACTATG	*ca*. 151 bp
	Reverse	TATCGGTTCTAGGACAGGCTCC	
AcMDB05	Forward	AACTGGGATTAGATACCCCACTATG	*ca*. 209 bp
	Reverse	GCTGGCGACGGTGGTATATA	
AcMDB07	Forward	GCCTATATACCGCCGTCG	*ca*. 321 bp
	Reverse	GTACACTTACCATGTTACGACTT	

^a^Thomsen et al. (2012); ^b^Shaw et al. (2016); ^c^Evans et al. (2015); ^d^DiBattista, Darren Coker, Stat, Michael Berumen, and Michael Bunce (2017); ^e^McInnes et al. (2017); ^f^Miya et al. (2015); ^g^Valentini et al. (2016).

**Figure 1:**
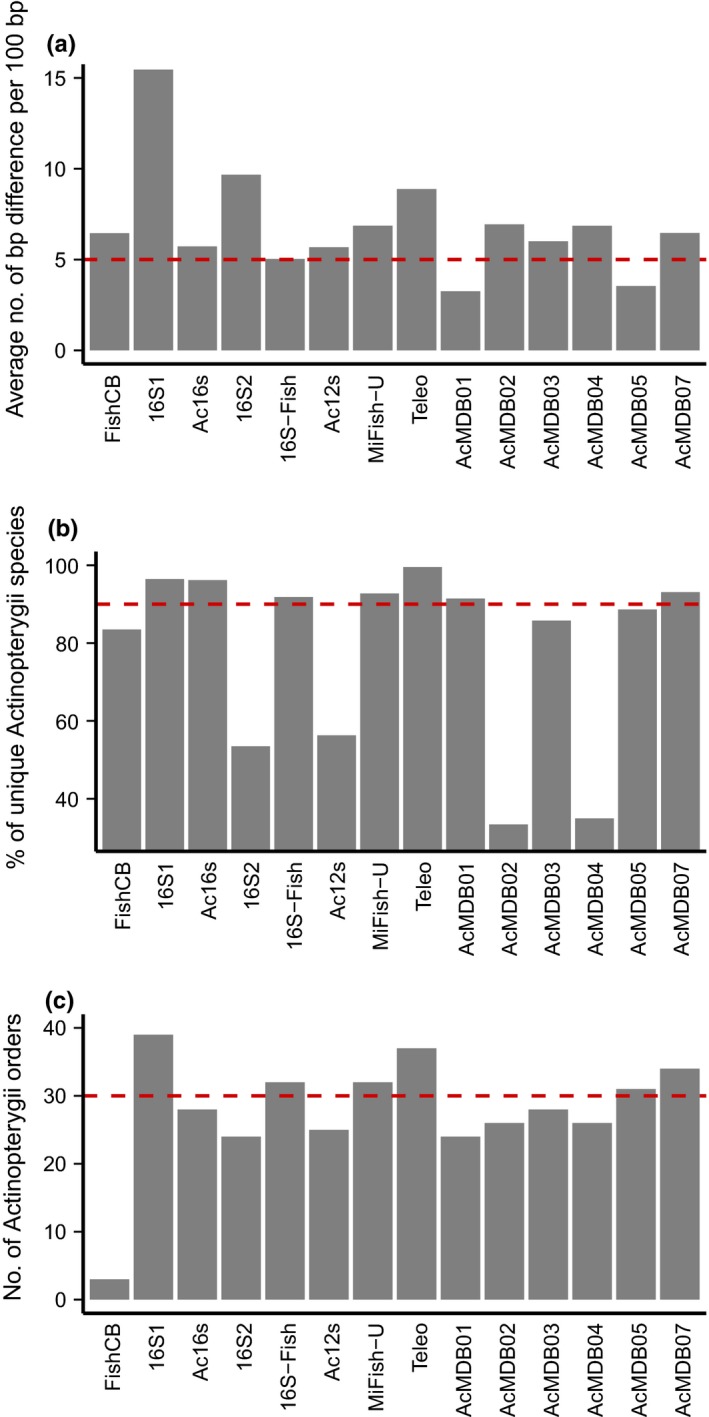
Summary statistics obtained from the initial screening of the primer pairs. Threshold values for each summary statistic are shown using a dashed red line. (a) The taxonomic resolution power of the barcodes is expressed as the average number of bp differences between species per 100 bases. (b) The specificity of the primer pairs is shown as the percentage of unique sequences belonging to *Actinopterygii* species. (c) The taxonomic coverage for each primer pair was evaluated as the number of Actinopterygii orders for which sequences were amplified in silico [Colour figure can be viewed at http://wileyonlinelibrary.com]

The summary statistics obtained from the initial primer screening are shown in Figure 1. Two, seven, and eight primer pairs fell below the threshold values set for the taxonomic resolution, primer specificity, and taxonomic coverage, respectively. When filtering primer pairs using all three summary statistics, five primers were deemed suitable for further analyses. Four primer pairs were obtained from previously published studies and are designed to amplify a fragment of the 16S (i.e., 16S1 and 16S‐Fish) and 12S (i.e., MiFish‐U and Teleo) mitochondrial gene (McInnes et al., 2017; Miya et al., 2015; Shaw et al., 2016; Valentini et al., 2016). Additionally, one of the newly developed primers (i.e., AcMDB07) passed the initial screening and amplifies an approximately 300 bp fragment of the 12S gene.

**Figure 2:**
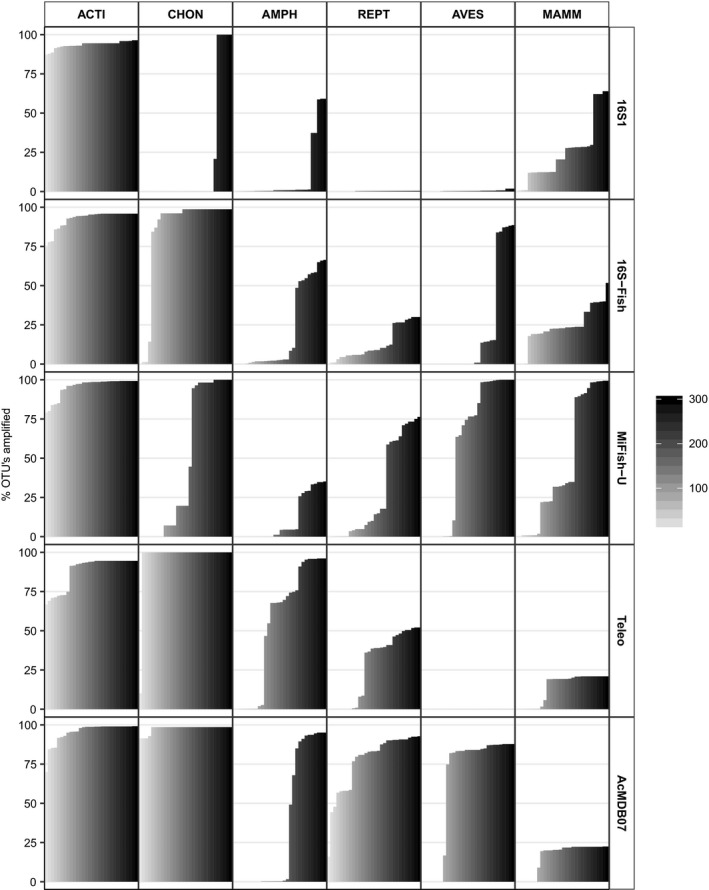
The estimated amplification success for all vertebrate classes and primer pairs. Amplification success was estimated using the R package PrimerMiner and threshold values ranging from 10 to 300 (i.e., light gray to black) with a stepwise increase of 10. Higher threshold values allow for more primer‐template mismatches thus leading to a higher amplification success. Amplification success was evaluated using sequence records from Actinopterygii (ACTI), Chondrichthyes (CHON), Amphibia (AMPH), Reptilia (REPT), Aves (AVES), and Mammalia (MAMM)

PrimerMiner analyses were used to evaluate the primer specificity and the impact of primer biases on an ecoregion scale and show clear differences for the different primers (Figure 2). The 16S1 primers appear highly specific to *Actinopterygii* species as high amplification success in the other vertebrate classes is only observed when high threshold values are used. The MiFish‐U primers are less specific as high amplification success is observed for Aves OTU's with midrange threshold values (i.e., 100–200). All other primer pairs successfully amplify Chondrichthyes OTU's even for low threshold values (i.e., <100). Successful amplification of other nontarget OUT's is also observed for high‐ (16S‐Fish primers) and midrange (Teleo and AcMDB07 primers) threshold values. When considering the amplification success within the Actinopterygii OUT's the results show that the Teleo primers are likely to suffer from primer biases as the amplification success is below 75% for threshold values below 90 and remains below 100% even for the highest threshold values (Figure 2). Although the 16S1 and 16S‐Fish primers have a higher amplification success for low threshold values, amplification success remains below 100% even for the highest threshold values. The MiFish‐U and AcMDB07 primers appear less prone to primer biases as high amplification success is achieved for low threshold values and amplification success approaches 100% for the higher threshold values.

The taxonomic resolution for all Actinopterygii families with occurrence records in the MDB and for all primer pairs is given in Table 3 and shows that the AcMDB07 primers offer the highest taxonomic resolution power. The 16S‐Fish and MiFish‐U primers also allow for high taxonomic assignments, while the barcoding regions amplified by the 16S1 and Teleo primers have the lowest taxonomic resolution (Table 3).

**Table 3:** The taxonomic resolution for all barcodes amplified by the different primers. Results are given as the percentage of sequences correctly identified to the genus and species level using a threshold of barcode similarity of 2 base pair (bp) and 5 bp for each primer pair

Primer ID	Threshold	Taxonomic resolution	
		Genus	Species
16S1	2	73.43	66.22
	5	51.10	38.22
16S‐Fish	2	83.14	72.90
	5	64.27	51.71
MiFish‐U	2	88.00	77.40
	5	69.88	55.48
Teleo	2	74.08	64.35
	5	52.80	38.90
AcMDB07	2	89.89	81.79
	5	77.90	64.45

### 3.2 Metabarcoding analyses

A total of 17,044,740 sequence reads were obtained from 243 uniquely labeled libraries resulting in an estimated average sequencing depth of *ca*. 70,000 reads per library. The overall quality of the run was low (Phred Q30 score ≥62.64) but this was not unexpected as amplicons with variable lengths will affect the quality of a run.

**Figure 3:**
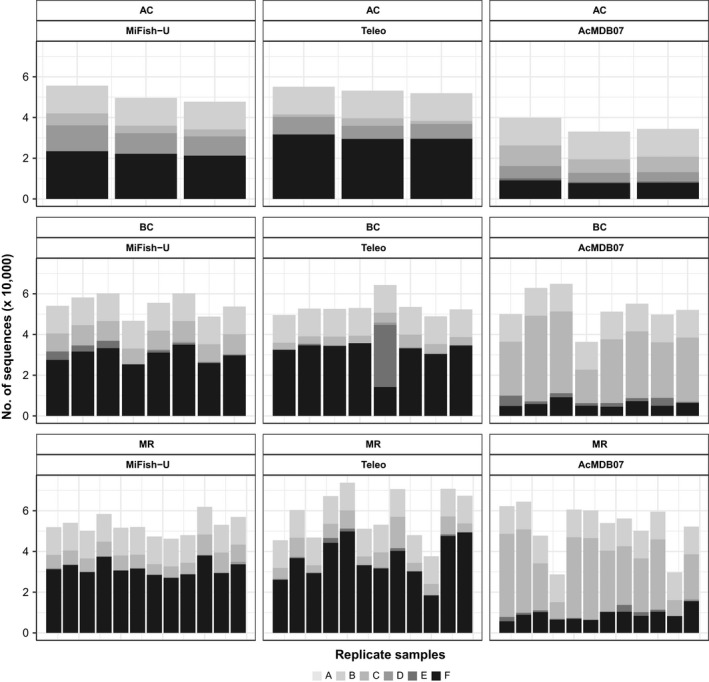
The number of sequence records removed during the bioinformatics filtering. Results are shown for the artificial community (AC) and the samples collected from Blakney Creek (BC) and the Murrumbidgee River (MR). The different gray scales represent the number of sequence reads removed when: (A) trimming the sequencing reads, (B) assigning sequencing reads to their respective samples, (C) removing short and low abundant sequence reads, (D) removing sequences with PCR and sequencing errors, and (E) assigning taxonomic information to the sequence reads (i.e., unassigned reads and non‐Actinopterygii reads). The sequence records assigned to *Actinopterygii* species for each sample are shown in black (F)

The effect of the bioinformatics filtering processes on the number of sequence reads was evaluated for all 96 amplicon libraries (Figure 3). While no obvious differences are observed in the number of sequence reads that passed the filtering process for the MiFish‐U and Teleo primers, the number of sequences assigned to *Actinopterygii* species for the AcMDB07 was substantially lower (Figure 3). Most of the sequence records obtained from the AcMDB07 primers were excluded when removing short and low abundant sequences (Figure 3). The sequence length distribution of all the reads that were assigned to their respective samples revealed that a relatively large number of sequences were shorter than 250 bp when using the AcMDB07 primers which were discarded during the bioinformatics filtering process (Appendix S1, Figure S1). Inspecting these short sequence records (i.e., BLAST search of 20 sequence records) revealed that the AcMDB07 primers amplify DNA of microorganisms although substantial bp mismatches are present between the primers and the amplified DNA fragments. To maximize the performance of the AcMDB07 primers, further optimization of the protocols is thus needed (see Section 4 for more details).

**Figure 4:**
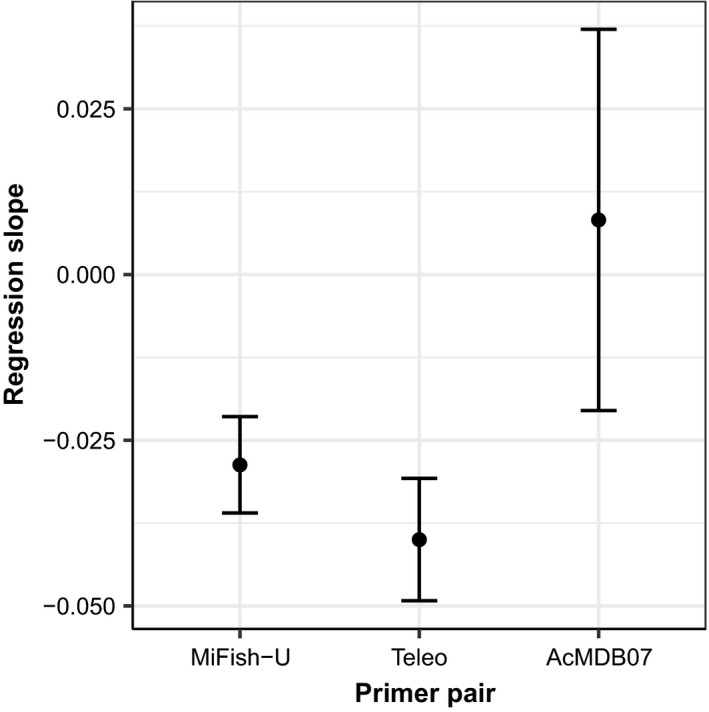
The estimated regression slopes for each primer pair. Regression slopes were estimated by fitting a linear mixed‐effect model to the proportional read abundance data, obtained from the artificial community sample, as a function of the PrimerMiner penalty scores. Solid points show the mean, and the error bars represent the 95% confidence interval around the mean

The estimates of the regression slope, obtained from fitting a linear mixed‐effect model to the logit‐transformed PRA data from the AC sample for each primer pair, show that there is a negative relationship between the PRA and the PrimerMiner penalty scores for both the MiFish‐U and Teleo primers (Figure 4 and Appendix S1, Figure S2). By contrast, the 95% confidence interval around the best estimate of the regression slope includes zero for the AcMDB07 primers, thus suggesting that primer‐template mismatches do not strongly influence amplification efficiency (Figure 4). However, it is important to recognize that the 95% confidence intervals for the AcMDB07 primers are quite large which is likely due to the low replication levels used here (i.e., only one artificial community was used).

**Figure 5:**
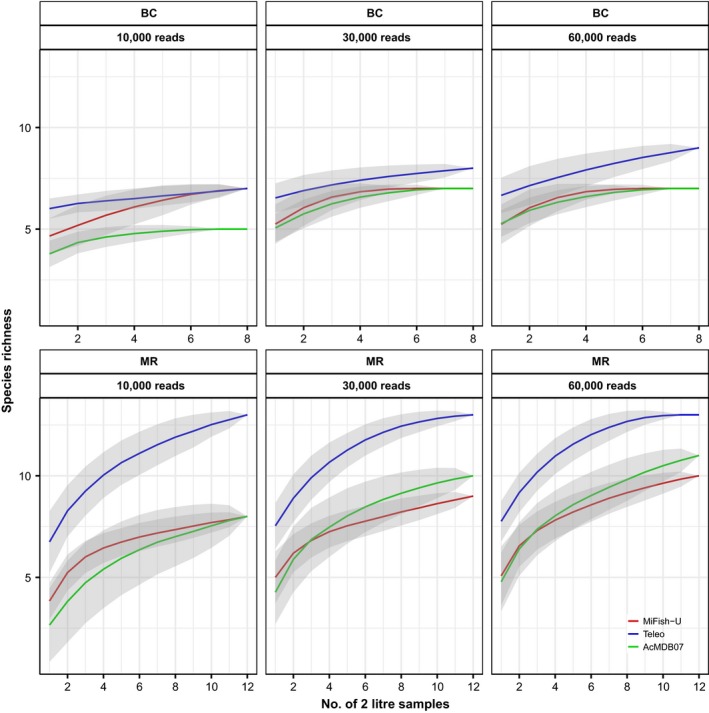
Species accumulation curves for the different primer pairs and the two sampling sites. Species accumulation curves are shown for the three different primer pairs (i.e., MiFish‐U, Teleo, and AcMDB07), the two different sampling sites (i.e., Blakney Creek [BC] and Murrumbidgee River [MR]), and the different levels of sequencing depth (i.e., 10,000; 30,000, and 60,000 reads per sample). The different levels of sequencing depth consider both Actinopterygii‐assigned reads and the reads discarded during the bioinformatics filtering process [Colour figure can be viewed at http://wileyonlinelibrary.com]

Species accumulations curves revealed that, in general, increasing the sampling intensity appears to have a more profound effect on the species richness than increasing the sequencing depth (Figure 5). While a sequencing depth of 10,000 reads per sample results in a noticeably lower species richness for the AcMDB07 primers, an increase in sequencing depth only moderately increases the species richness for the MiFish‐U and Teleo primers (Figure 5). The Teleo primers detected the highest number of fish species, and the difference between the species accumulation curves of the Teleo primers and the MiFish‐U and AcMDB07 primers is more pronounced for the MR sampling site then for the BC sampling site. No strong differences are observed for the curves obtained with the MiFish‐U and AcMDB07 primers (Figure 5).

**Figure 6:**
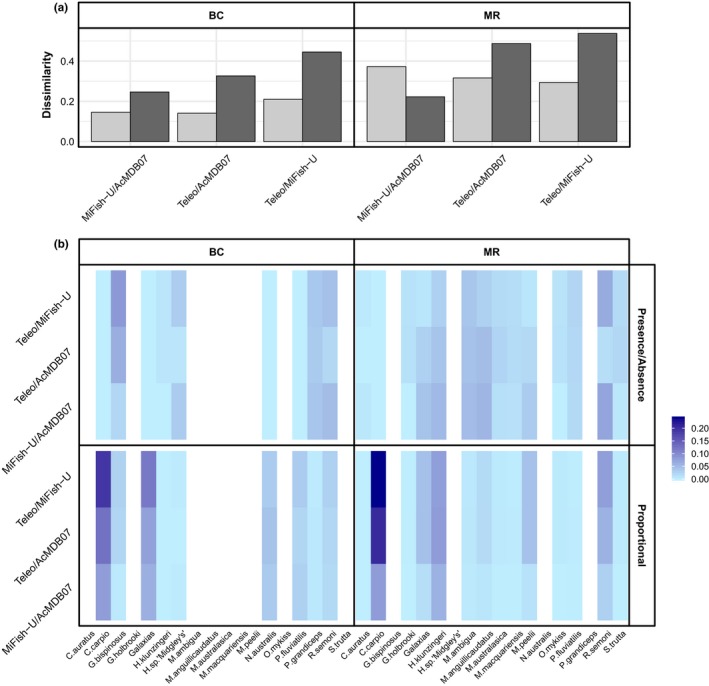
The results of the community dissimilarity analyses for the different primer pairs. The results show the overall dissimilarity between the fish community data obtained using the different primer pairs (a), and the heat map shows the average contribution of each species to the overall dissimilarity (b). The community dissimilarity was evaluated for both the Blakney Creek (BC) and the Murrumbidgee River (MR) sampling sites using the presence/absence community data (light gray bars in plot [a] and upper panels in plot [b]) and proportional abundance data (dark gray bars in plot [a] and lower panels in plot [b]) [Colour figure can be viewed at http://wileyonlinelibrary.com]

Primer pairs have a significant effect on the fish community data obtained from the BC and MR sampling sites, in terms of the presence/absence data and proportional abundance data (*p*‐values <0.05). The community dissimilarity between the different primer pairs is generally higher for the proportional abundance data (Figure 6a). The only exception to this pattern was the comparison between the MiFish‐U and AcMDB07 primers for the MR site. Another pattern evident from the results is that the proportional abundance data obtained from the Teleo primers showed higher dissimilarity with the MiFish‐U and AcMDB07 primers than the dissimilarity between the MiFish‐U and AcMDB07 primers (Figure 6a). When evaluating the average contribution of each species to the overall dissimilarity, clear differences are observed between the presence/absence and the proportional abundance data (Figure 6b). The relative abundance of *Cyprinus carpio* sequence reads has a substantial contribution to the community dissimilarity with the Teleo primers showing consistently lower proportional read abundances compared to both the MiFish‐U and AcMDB07 primers (Figure 6b; Appendix S1, Table S3). Additionally, the relative read abundance of *Galaxias* sp. seems to be an important driver for the community dissimilarities in the BC site. For the MR site, the relative abundance of *Hypseleotris klunzingeri* and *Retropinna semoni* sequences varies between primers. In contrast, for the presence/absence community data of the BC site *Gadopsis bispinosus*,* Hypseleotris* sp. *“Midgley's carp gudgeon”*,* Philypnodon grandiceps,* and *R. semoni* explain most of the community‐level variation between the different primer pairs (Figure 6b). The presence/absence of *Galaxias* sp., *H. klunzingeri*,* Macquaria ambigua*,* Misgurnus anguillicaudatus,* and *R. semoni* sequences account for most of the observed community variation within the MR site (Figure 6b).

## 4 DISCUSSION

The in silico workflow presented here allows for a robust evaluation of metabarcoding primers and reveals that different primers have different advantages and disadvantages. Our results reveal that different trade‐offs need to be considered when choosing the optimal primer pair for eDNA metabarcoding surveys. While the 16S1 primers are highly specific to *Actinopterygii* species, these primers are likely to suffer from primer biases (Figure 2). The 16S‐Fish, Teleo, and AcMDB07 primers are less specific as amplification of Chondrichthyes OTU's is observed. While this nontarget amplification is less of an issue for freshwater metabarcoding surveys, the ability to amplify *Chondrichthyes* species with these primer pairs could make them more suitable for marine metabarcoding surveys focussing on the entire fish biodiversity (i.e., *Actinopterygii* and *Chondrichthyes* species). Only two primer pairs (MiFish‐U and AcMDB07) achieve 100% amplification success for the Actinopterygii OTU's in silico (Figure 2). Thus, amplification biases due to primer‐template mismatches are predicted to be less problematic for the MiFish‐U and AcMDB07 primers. Other primers are predicted to be more affected by amplification biases, and certain species may even remain undetected due to high primer‐template mismatches (i.e., amplification success of Actinopterygii OTU's is below 100% even when high threshold values are used; Figure 2). In addition, the barcoding regions amplified by the MiFish‐U and AcMDB07 primers provided the highest taxonomic assignment power (Table 3) and these primer pairs are thus predicted to be most suitable for eDNA metabarcoding surveys in the MDB.

Ideally, a thorough in vitro evaluation of the presented workflow should utilize eDNA samples representative of the entire ecoregion and all five metabarcoding primers which passed the initial screening should be tested. However, due to financial constraints, we only used the primers targeting the 12S mitochondrial gene to validate the newly developed primer pair and assess the performance of our in silico workflow. Additionally, one of the primer pairs used in the in silico evaluation has previously been used for eDNA metabarcoding surveys within the MDB (i.e., 16S1; Shaw et al., 2016). The results presented by Shaw et al. (2016) revealed that the 16S1 primers detected only a limited number of species compared to a general vertebrate primer. While the authors recognize that the absence of reference sequences for some species may explain their findings, our analyses show that high primer‐template mismatches for some species are also likely to affect the performance of this primer pair. The in silico analyses and the data obtained from the AC both show that the Teleo primers are more strongly affected by amplification biases compared to the MiFish‐U and AcMDB07 primers (Figures 2 and 4). However, despite the general belief that an increase in primer‐template mismatches will reduce species detections, the results of the eDNA samples showed that more fish species are detected with the Teleo primers (Figure 5). This somewhat counterintuitive observation can be explained when taking into consideration the high average contribution of common carp (*Cyprinus carpio*) to the community dissimilarity when using the proportional read data (Figure 6b). Overall, the proportion of carp sequences is much lower for the Teleo primers (Appendix S1, Table S3). Common carp is a highly successful invasive fish in the MDB and carp biomass can make up 70–90 percent of the total fish biomass (Koehn, 2004; Lintermans, 2007). Given that carp is known to be highly abundant in both sampling sites, the proportional read abundances from the MiFish‐U and AcMDB07 primers may better reflect the actual community composition. The lower proportion of carp sequences for the Teleo primers is likely to be the results of a reduced amplification efficiency as the in silico analyses revealed a higher penalty score for the Teleo primers and common carp sequence compared to the MiFish‐U and AcMDB07 primers (i.e., penalty scores were 84.9, 6.2, and 18.45 for the respective primers). The reduced amplification efficiency of carp eDNA with the Teleo primers is thus likely to reduce the swamping effect from a single species which is a commonly encountered issue in DNA‐based dietary analyses (Shehzad et al., 2012; Vestheim & Jarman, 2008). Otherwise, the shorter barcoding region amplified by the Teleo primers could also increase the detection of fish taxa due to an increased ability to recover highly degraded eDNA. Although recent studies have suggested that the aquatic environment may preserve eDNA relatively well (Bylemans et al., 2018; Piggott, 2016), more research is needed to evaluate the effect of barcode length on eDNA metabarcoding surveys. Overall, the results show that even within an ecoregion the performance of eDNA metabarcoding primers may differ depending on the local biodiversity. Within the MDB, the Teleo primers may recover more species in systems dominated by common carp. However, the MiFish‐U and AcMDB07 primers do provide a higher taxonomic resolution and will thus provide more accurate species‐level assignments. Thus, any prior information on the local biodiversity (e.g., obtained from conventional surveys) and the aim of the metabarcoding survey will need to be carefully considered to determine the most suitable primer pair.

Although an in silico evaluation of metabarcoding primers can help guide primer selection, it is important to consider and discuss the limitations. First, the availability and quality of reference sequences will affect the ability to design “universal” primers and can affect the performance of computer‐based simulations (Elbrecht & Leese, 2016). The lack of appropriate reference sequences is a well‐known issue for eDNA metabarcoding surveys and is particularly problematic in ecoregions with a high number of endemic species. Thus, the design and performance evaluation of metabarcoding primers will benefit from custom databases with taxonomically verified records and/or complementing publicly available databases with de novo generated sequences. Second, evaluating amplification biases due to primer‐template mismatches is impossible when reference databases are generated solely for the barcoding region of interest (Valentini et al., 2016). Reference sequences consisting of the entire gene of interest on the other hand will ensure that the impact of primer‐template mismatches can be assessed and will increase the versatility of the reference database. In addition, amplification biases can also arise from different starting concentrations of template DNA in the eDNA extracts. The results from our primer validation study show that the impact of the relative starting concentrations of eDNA will differ depending on the primer pair. While estimates of the relative abundance of different species and a thorough understanding of the primer‐template mismatches may help in the selection of the most suitable primer pair, pilot studies will remain invaluable to fully evaluate the performance of metabarcoding primers.

At last, the newly developed AcMDB07 primers are suitable for eDNA metabarcoding applications. The results from the AC show that the AcMDB07 primers are not strongly affected by amplification biases due to primer‐template mismatches (i.e., regression slope ≈ 0; Figure 4). Thus, this primer pair may be more suitable to obtain (semi‐) quantitative data from eDNA metabarcoding surveys (Elbrecht & Leese, 2015; Pinol et al., 2015). The primer validation based on the eDNA samples also revealed that the AcMDB07 primers detect a similar number of species compared to the MiFish‐U primers (Figure 5). It is, however, important to note that the amplification of DNA from microorganisms by the AcMDB07 primers is a potential concern and protocol modifications are likely to improve the performance of this primer pair. Increasing the annealing temperature during the PCR amplification may help increase the specificity of the AcMDB07 primers but could also increase the impact of amplification biases (Clarke et al., 2017; Pinol et al., 2015). A more appropriate size selection protocol prior to HTS can also eliminate unwanted amplicons from the library and will increase the sequencing depth of the desired amplicons. For the AcMDB07 primers, this can be achieved by reducing the volume ratio of Agencourt AMPure XP Beads to 0.8 to remove all amplicons shorter than 200 bp (Appendix S1, Figure S1). For the future use of the AcMDB07 primers, we recommend a combined approach with a slight increase in annealing temperatures (e.g., 55°C) and the use of a more stringent size selection protocol during the library cleanup.

## 5 CONCLUSION

The in silico workflow presented here allows for a robust evaluation of metabarcoding primers and can be easily transferred to other ecoregions and other taxonomic groups. As the use of group‐specific metabarcoding primers is likely to increase in the future, computer‐based simulations will become increasingly valuable in order to make well‐informed decisions on the most suitable primer pairs for the study region of interest.

## CONFLICT OF INTEREST

None declared.

## AUTHOR CONTRIBUTION

J.B., E.M.F., C.M.H., and D.M.G. designed the study. J.B. performed all field work, laboratory work, and all data analyses. All authors contributed to the writing of the manuscript.

## DATA ACCESSIBILITY

All de novo generated sequences are available on GenBank (accession numbers: KY798443‐KY798504). The R script used to perform the in silico analyses is available as Supporting Information (Appendix S2). The summarized metabarcoding data and the R script used to analyze the data are also available as Supporting Information (Appendix S3).
