# Supplementary material for: Toward an ecoregion scale evaluation of eDNA metabarcoding primers: A case study for the freshwater fish biodiversity of the Murray–Darling Basin (Australia)
Source: Ecol Evol. 2018 Aug 5;8(17):8697–712. doi: 10.1002/ece3.4387 (PMC6157654; doi:10.1002/ece3.4387)
Supplement: Supplementary file 1 [file ECE3-8-8697-s001.pdf]

Towards an ecoregion scale evaluation of eDNA metabarcoding primers: a case-study for the freshwater fish biodiversity of the Murray-Darling Basin (Australia)

Jonas Bylemans[1, 2*], Dianne M. Gleeson[1, 2], Christopher M. Hardy[3, 2], Elise Furlan[1, 2]

[1] Institute for Applied Ecology, University of Canberra, Canberra, ACT, Australia

[2] Invasive Animals Cooperative Research Centre, University of Canberra, Canberra, ACT 2617, Australia

[3] CSIRO Land and Water, GPO Box 1700, Canberra, ACT, Australia

* Corresponding author: Jonas.Bylemans@canberra.edu.au

Contents

Amplification and sequencing of the 12S ribosomal RNA gene for all freshwater fish species of the Murray-Darling Basin (MDB). 4

List of vertebrate families with occurence records in the MDB. 10

Sequence lenght distribution of all sequence reads assigned to their respective samples. 14

Best fitting linear regression model for the eDNA metabarcoding data obtained from the artificial community sample. 15

Summary of the metabarcoding data obtained from environmental DNA samples collected from two sites in the MDB. 16

References 18

Tables

Table S1. Complete list of all freshwater fish species the Murray-Darling Basin (MDB) and the details of all the samples used for the PCR amplification and Sanger sequencing of the entire mitochondrial 12S ribosomal RNA gene (NCBI accession codes: KY798443-KY798504). 6

Table S2. List of all major vertebrate families with occurrence records in the Darling River Drainage (Atlas of Living Australia). 10

Table S3. Summary of the metabarcoding data obtained from environmental DNA samples collected for two sites within the Murray-Darling Basin (i.e. 8 and 12 samples collected for the Blakney Creek and Murrumbidgee River sites respectively) and analysed with the MiFish-U, Teleo and AcMDB07 primer pairs. Results are given as the number of samples testing positive for the different species and the average proportion of sequence reads ± the standard deviation given in between brackets. 16

Figures

Figure S1. The number of internally amplified barcodes for each primer pair plotted against the length of the internal barcode sequences. The data are derived from all sequence records that were successfully assigned to their respective samples and the vertical dashed lines represent the sequence length threshold used to remove short sequence records for each primer pair. 14

Figure S2. The best fitting model describing the relationship between the proportional read abundances and the PrimerMiner penalty scores for the artificial community. 15

Amplification and sequencing of the 12S ribosomal RNA gene for all freshwater fish species of the Murray-Darling Basin (MDB).

A genetic database for all freshwater Actinopterygii species with established populations in the MDB was obtained using a PCR amplification of the complete mitochondrial 12S ribosomal RNA gene followed by Sanger sequencing. For all species, either extracted DNA or tissue samples were obtained from previous studies (Hardy et al., 2011; MacDonald, Young, Lintermans, & Sarre, 2014) (Table S1). When only tissues samples were available, genomic DNA was extracted using the DNeasy Blood and Tissue Kit following the manufacturer instructions (Qiagen, Hilden, Germany).

For most samples, successful amplification of the entire 12S gene was achieved using primer combinations 12SR and 12SL or Marinefish-12SrRNA-F and Marinefish-12SrRNA-R (Jin, Zhao, & Wang, 2013; Wang, Tsai, Tu, & Lee, 2000). PCR reactions contained 12.50 µL MyTaq^{TM} HS Red Mix (Bioline Australia Pty Ltd, NSW, Australia), 0.25-1.00 µL of each primer (10µM), 1.00-4.00 µL genomic DNA and DEPC-treated water to a final volume of 25 µL. Cycling conditions consisted of an initial activation of 2 min at 95°C; 35 3-step cycles of 1 min at 94°C, 1 min at 50°C and 1 min 30 sec at 72°C; and a final extension of 10 min at 72°C. For three species (i.e. *Galaxias ornatus*, *Maccullochella peelii* and *Pseudaphritis urvillii*) modifications to the PCR protocol were needed. For *G. ornatus* and *M. peelii* the 12SV5 primers described by Riaz et al. (Riaz et al., 2011) were used as internal PCR primers in combination with Marinefish-12S-F and 12SR. Additionally, a touchdown cycling stage (i.e. 10 3-step cycles of 1 min at 94°C, 1 min at 60°C and 1 min 30 sec at 72°C with annealing temperatures decreasing with 1°C per cycle) was added after the initial activation step to increase specificity and yield. Successful amplification of the 12S gene of *P. urvillii* required

newly developed primers Not-12S-F (5’-TATTTAAAACGTAACACTGAAAATG-3’) and Not-12S-R (5’-TCATGATGCAAAAGGTACGAG-3’) as previously used primers contained significant base-pair mismatches with sequence records of other species within the suborder Notothenioidei.

The presence of a single PCR product was confirmed through gel electrophoresis using a 2% agarose gel containing SYBR® Safe DNA gel stain and a run time of 60 min at 90 volts. Amplicons were purified using the MinElute PCR Purification Kit (Qiagen, Hilden, Germany) and Sanger sequenced using an AB 3730xl DNA Analyzer at the ACRF Biomolecular Resource Facility (The John Curtin School of Medical Research, Australian National University). PCR primers were used for sequencing and an internal sequencing primer (MT1478H) was used to improve sequencing quality of the 5’ region of the 12S gene for most samples (excluding *G. ornatus* and *M. peelii*) (Fuller, Baverstock, & King, 1998). Sequences were imported into Geneious v8.1.8 and assembled into contigs using the “DeNovo Assembly” option (Kearse et al., 2012). Assemblies were manually checked for quality and a consensus sequence was obtained containing a partial sequence of the Phenylalanyl-tRNA gene, the whole 12S ribosomal RNA gene and Valine-tRNA gene, and a partial sequence of the 16S ribosomal RNA gene (NCBI accession codes: KY798443-KY798504).

Table S1. Complete list of all freshwater fish species the Murray-Darling Basin (MDB) and the details of all the samples used for the PCR amplification and Sanger sequencing of the entire mitochondrial 12S ribosomal RNA gene (NCBI accession codes: KY798443-KY798504).

Species Name	Origin	Isolate	Source
Afurcagobius tamarensis	Native	CES-088	Donovans Landing (SA); Hardy et al. (2011)
Ambassis agassizii	Native	CES-224	Brewster Outlet Channel (NSW); Hardy et al. (2011)
Anguilla australis	Native	CES-080	Onkaparinga River (SA); Hardy et al. (2011)
Anguilla reinhardtii	Native	CES-064	Seafood Trade (VIC); Hardy et al. (2011)
Atherinosoma microstoma	Native	CES-084	Mundoo Channel (SA); Hardy et al. (2011)
Bidyanus bidyanus	Native	CES-043	Narrandera Fisheries Centre (NSW); Hardy et al. (2011)
Carassius auratus	Invasive	CES-025	Narrandera Fisheries Centre (NSW); Hardy et al. (2011)
Carassius carassius	Invasive	TR-1709	Campaspe River (VIC); Raadik T.A.
Craterocephalus amniculus	Native	CES-675	Gwydir River (NSW); Hardy et al. (2011)
Craterocephalus fluviatilis	Native	CES-007	Cardross Lakes (VIC); Hardy et al. (2011)
Craterocephalus stercusmuscarum fulvus	Native	CSF9934.1	Murray River (NSW); Unmack P.J.
Cyprinus carpio	Invasive	CES-005	Lower Torrens River (SA); Hardy et al. (2011)
Gadopsis bispinosus	Native	CES-009	Cotter River (ACT); Hardy et al. (2011)
Gadopsis marmoratus	Native	CES-016	LaTrobe River (VIC); Hardy et al. (2011)

Galaxias arcanus	Native	CES-021	King River (VIC); Hardy et al. (2011)
Galaxias brevipinnis	Native	CES-010	Victoria Creek (SA); Hardy et al. (2011)
Galaxias fuscus	Native	CES-022	Plain Creek (VIC); Hardy et al. (2011)
Galaxias maculatus	Native	CES-087	Lower Myponga River (SA); Hardy et al. (2011)
Galaxias olidus	Native	CES-019	Lachlan River (NSW); Hardy et al. (2011)
Galaxias oliros	Native	CES-023	King River (VIC); Hardy et al. (2011)
Galaxias ornatus	Native	TR-4399	Hirts Creek (VIC); Raadik T.A.
Galaxias rostratus	Native	CES-024	Goulburn River (VIC); Hardy et al. (2011)
Galaxias tantangara	Native	TR-4382	Tantangara Creek (NSW); Raadik T.A.
Galaxias truttaceus	Native	CES-079	McIvor River (VIC); Hardy et al. (2011)
Gambusia holbrooki	Native	CES-026	Narrandera Fisheries Centre (NSW); Hardy et al. (2011)
Geotria australis	Native	CES-082	Goolwa Barrage (SA); Hardy et al. (2011)
Hypseleotris klunzingeri	Native	CES-003	Murray River (VIC); Hardy et al. (2011)
Hypseleotris sp.1 "midgley's"	Native	CES-030	Calperum (SA); Hardy et al. (2011)
Hypseleotris sp.2 "Lake"	Native	HLak3	Black Swamp (VIC); Unmack P.J.
Hypseleotris sp.3 "murray-darling"	Native	CES-034	Dunns Swamp (NSW); Hardy et al. (2011)
Leiopotherapon unicolor	Native	CES-264	Caliguel Lagoon (QLD); Hardy et al. (2011)

Maccullochella macquariensis	Native	UC0524	Bendora Reservoir (ACT); MacDonald et al. (2014)
Maccullochella peelii	Native	MP-#28	Murrumbidgee River (ACT); Couch A.J.
Macquaria ambigua ambigua	Native	GPMB.1	Murray River (SA); Unmack P.J.
Macquaria australasica	Native	CES-208	Cotter River (ACT); Hardy et al. (2011)
Melanotaenia fluviatilis	Native	CES-028	Murray/Darling confluence (NSW); Hardy et al. (2011)
Melanotaenia splendida tatei	Native	CES-029	Paroo River (QLD); Hardy et al. (2011)
Misgurnus anguillicaudatus	Invasive	CES-074	Murrumbidgee River (ACT); Hardy et al. (2011)
Mogurnda adspersa	Native	CES-006	Murray Bridge (SA); Hardy et al. (2011)
Mordacia mordax	Native	CES-083	Goolwa Barrage (SA); Hardy et al. (2011)
Nannoperca australis	Native	CES-012	Finniss River (SA); Hardy et al. (2011)
Nannoperca obscura	Native	CES-011	Finniss River (SA); Hardy et al. (2011)
Nematalosa erebi	Native	CES-002	Lake Alexandrina (SA); Hardy et al. (2011)
Neosilurus hyrtlii	Native	CES-001	Warrego River (QLD); Hardy et al. (2011)
Oncorhynchus mykiss	Invasive	CES-077	Eucumbene Trout Farm (NSW); Hardy et al. (2011)
Oxyeleotris lineolata	Native	CES-232	Aquarium Trade (ACT); Hardy et al. (2011)
Perca fluviatilis	Invasive	CES-004	Murray River (SA); Hardy et al. (2011)
Percalates colonorum	Native	CES-040	Snowy River (VIC); Hardy et al. (2011)

Percalates novemaculeata	Native	CES-231	Aquarium Trade (ACT); Hardy et al. (2011)
Philypnodon gandiceps	Native	CES-014	Martins Bend Wetland (SA); Hardy et al. (2011)
Philypnodon macrostomus	Native	CES-013	Martins Bend Wetland (SA); Hardy et al. (2011)
Porochilus rendahli	Native	CES-170	Beardmore Dam (QLD); Hardy et al. (2011)
Pseudaphritis urvillii	Native	CES-085	Mundoo Channel (SA); Hardy et al. (2011)
Pseudogobius olorum	Native	CES-081	Finniss River (SA); Hardy et al. (2011)
Retropinna semoni	Native	CES-035	Martins Bend Wetland (SA); Hardy et al. (2011)
Rutilus rutilus	Invasive	CES-075	Moorabool River (VIC); Hardy et al. (2011)
Salmo salar	Invasive	CES-070	Seafood Trade (ACT); Hardy et al. (2011)
Salmo trutta	Invasive	CES-069	Gellibrand River (VIC); Hardy et al. (2011)
Salvelinus fontinalis	Invasive	CES-071	Eucumbene Trout Farm (NSW); Hardy et al. (2011)
Tandanus tandanus	Native	CES-050	Namoi River (NSW); Hardy et al. (2011)
Tasmanogobius lasti	Native	CES-086	Lake Bonney (SA); Hardy et al. (2011)
Tinca tinca	Invasive	CES-076	Campaspe River (VIC); Hardy et al. (2011)

List of vertebrate families with occurence records in the MDB.

Table S2. List of all major vertebrate families with occurrence records in the Darling River Drainage (Atlas of Living Australia).

Class	Order	Family
Actinopterygii	Anguilliformes	Anguillidae
	Clupeiformes	Clupeidae
	Galaxiiformes	Galaxiidae
	Osmeriformes	Retropinnidae
	Siluriformes	Plotosidae
	Atheriniformes	Atherinidae; Melanotaeniidae
	Perciformes	Ambassidae; Bovichtidae; Percidae
	Centrarchiformes	Percichthyidae; Terapontidae
	Gobiiformes	Eleotridae; Gobiidae
	Salmoniformes	Salmonidae
	Cypriniformes	Cyprinidae; Cobitidae
	Cyprinidontiformes	Poeciliidae
Chondrichthyes	Carcharhiniformes	Triakidae; Sphyrnidae; Carcharhinidae; Scyliorhinidae

	Lamniformes	Lamnidae; Mitsukurinidae; Odontaspididae
	Pristiophoriformes	Pristiophoridae
	Myliobatiformes	Myliobatidae; Dasyatidae
	Chimaeriformes	Callorhinchidae
	Hexanchiformes	Hexanchidae
	Orectolobiformes	Orectolobidae
	Heterodontiformes	Heterodontidae
Amphibia	Anura	Myobatrachidae; Hylidae; Bufonidae; Microhylidae
Reptilia	Squamata	Scincidae; Agamidae; Diplodactylidae; Elapidae; Gekkonidae; Pygopodidae; Varanidae; Typhlopidae; Carphodactylidae; Boidae; Colubridae; Acrochordidae
	Testudines	Chelidae; Cheloniidae; Dermochelyidae
	Crocodylia	Crocodylidae
Aves	Passeriformes	Meliphagidae; Artamidae; Acanthizidae; Pachycephalidae; Rhipiduridae; Monarchidae; Corvidae; Pardalotidae; Maluridae; Petroicidae; Sturnidae; Hirundinidae; Campephagidae; Climacteridae; Passeridae; Corcoracidae; Estrildidae; Timaliidae; Turdidae; Pomatostomidae; Megaluridae; Nectariniidae; Motacillidae; Fringillidae; Oriolidae; Oreoididae; Neosittidae; Acrocephalidae; Ptilonorhynchidae; Psophodidae; Alaudidae; Cisticolidae; Menuridae; Dasyornithidae; Dicruridae; Orthonychidae; Pittidae; Paradisaeidae; Pycnonotidae; Atrichornithidae; Ploceidae; Sylviidae
	Psittaciformes	Psittacidae; Cacatuidae

Anseriformes	Anatidae; Anseranatidae
Falconiformes	Accipitridae; Falconidae
Charadriiformes	Charadriidae; Laridae; Scolopacidae; Recurvirostridae; Burhinidae; Haematopodidae; Pedionomidae; Glareolidae; Rostratulidae; Jacanidae; Stercorariidae
Columbiformes	Columbidae
Ciconiiformes	Threskiornithidae; Ardeidae; Ciconiidae
Pelecaniformes	Phalacrocoracidae; Pelecanidae; Anhingidae; Phaethontidae; Sulidae; Fregatidae
Coraciiformes	Alcedinidae; Meropidae; Coraciidae
Gruiformes	Rallidae; Gruidae; Otididae
Cuculiformes	Cuculidae; Centropodidae
Podicipediformes	Podicipedidae
Strigiformes	Strigidae; Tytonidae
Struthioniformes	Casuariidae; Struthionidae
Galliformes	Phasianidae; Megapodiidae; Numididae
Apodiformes	Aegothelidae; Apodidae
Caprimulgiformes	Podargidae; Caprimulgidae
Turniciformes	Turnicidae

	Procellariiformes	Procellariidae; Diomedeidae; Oceanitidae
	Sphenisciformes	Spheniscidae
	Accipitriformes	Accipitridae
Mammalia	Diprotodontia	Macropodidae; Phalangeridae; Vombatidae; Pseudocheiridae; Phascolarctidae; Petauridae; Potoroidae; Burramyidae; Acrobatidae; Hypsiprymnodontidae
	Chiroptera	Vespertilionidae; Molossidae; Miniopteridae; Pteropodidae; Emballonuridae; Rhinolophidae; Rhinonycteridae; Megadermatidae; Hipposideridae
	Carnivora	Canidae; Felidae; Otariidae; Mustalidae; Phocidae
	Rodentia	Muridae
	Dasyuromorphia	Dasyuridae; Myrmecobiidae
	Lagomorpha	Leporidae
	Artiodactyla	Bovidae; Cervidae; Suidae; Camelidae
	Monotremata	Tachyglossidae; Ornithorhynchidae
	Peramelemorphia	Peramelidae; Thylacomyidae; Chaeropodidae
	Perrisodactyla	Equidae
	Cetacea	Delphinidae; Balaenidae; Physeteridae; Ziphiidae; Phocoenidae; Balaenopteridae; Neobalaenidae; Kogiidae
	Sirenia	Dugongidae

Sequence lenght distribution of all sequence reads assigned to their respective samples.

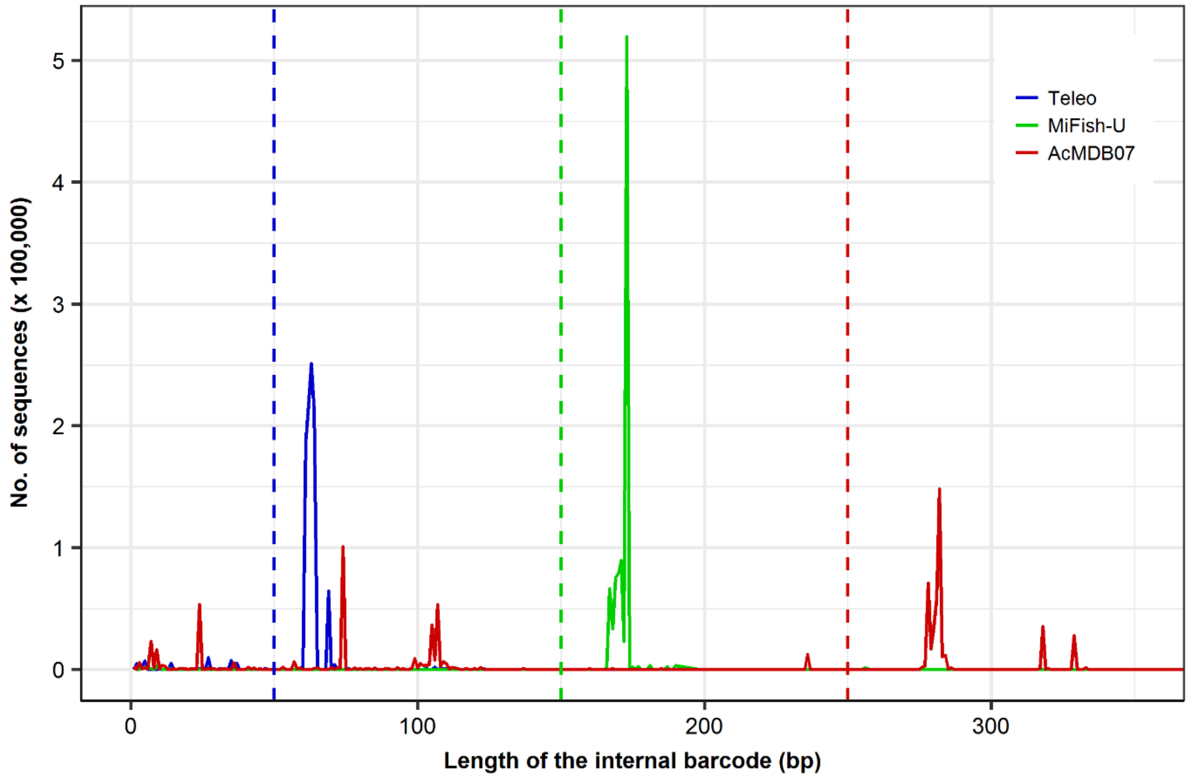

Figure S1. The number of internally amplified barcodes for each primer pair plotted against the length of the internal barcode sequences. The data are derived from all sequence records that were successfully assigned to their respective samples and the vertical dashed lines represent the sequence length threshold used to remove short sequence records for each primer pair.

Best fitting linear regression model for the eDNA metabarcoding data obtained from the artificial community sample.

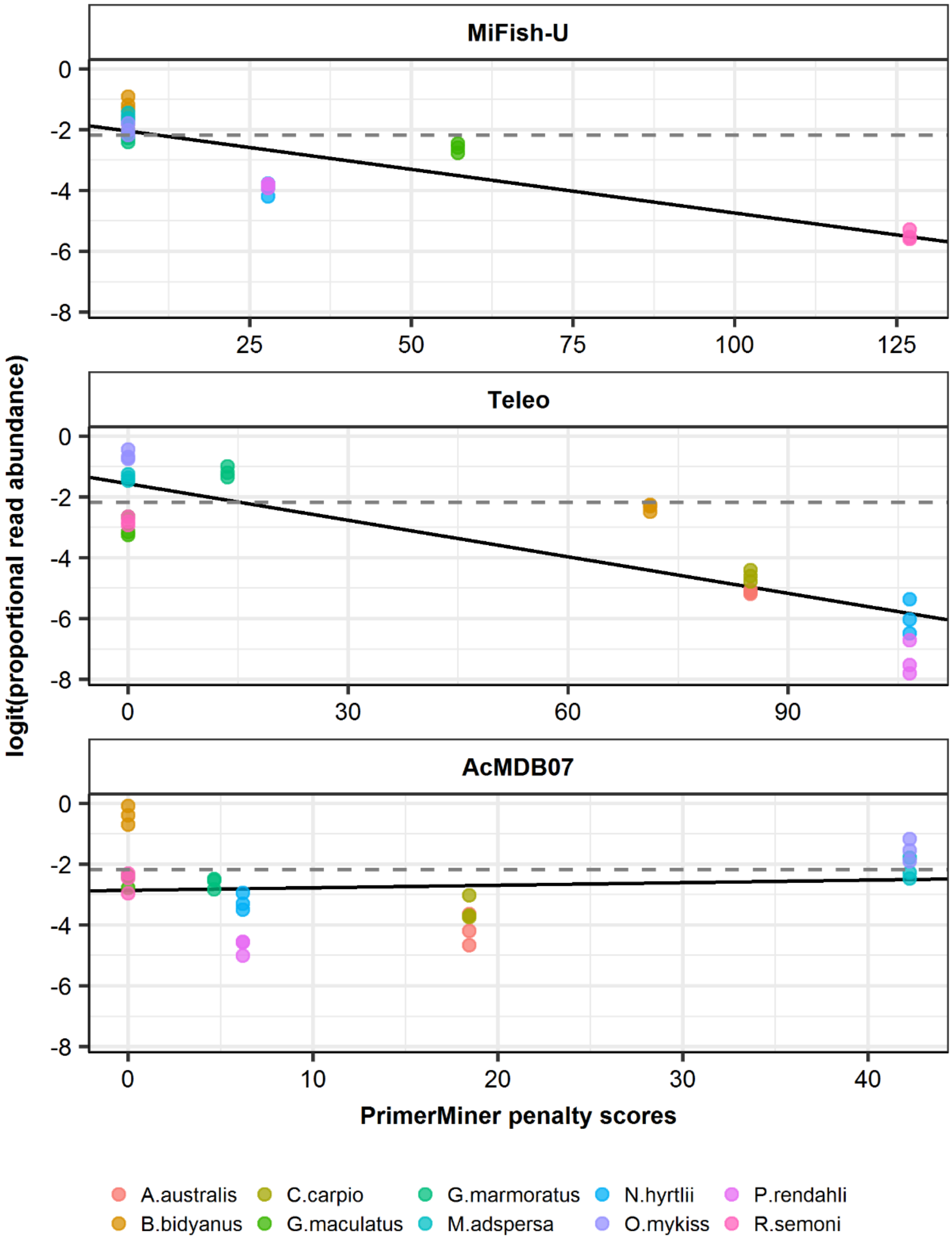

Figure S2. The best fitting model describing the relationship between the proportional read abundances and the PrimerMiner penalty scores for the artificial community.

Summary of the metabarcoding data obtained from environmental DNA samples collected from two sites in the MDB.

Table S3. Summary of the metabarcoding data obtained from environmental DNA samples collected for two sites within the Murray-Darling Basin (i.e. 8 and 12 samples collected for the Blakney Creek and Murrumbidgee River sites respectively) and analysed with the MiFish-U, Teleo and AcMDB07 primer pairs. Results are given as the number of samples testing positive for the different species and the average proportion of sequence reads ± the standard deviation given in between brackets.

**Species name**	**Blakney Creek**			**Murrumbidgee River**		
	MiFish-U	Teleo	AcMDB07	MiFish-U	Teleo	AcMDB07
C. auratus	0	0	0	1 (0.004)	0	0
C. carpio	8 (0.448 ± 0.074)	8 (0.064 ± 0.019)	8 (0.317 ± 0.118)	12 (0.847 ± 0.134)	12 (0.369 ± 0.132)	12 (0.757 ± 0.175)
G. bispinosus	0	8 (0.057 ± 0.036)	2 (0.044 ± 0.009)	0	0	0
G. holbrooki	0	0	0	0	2 (0.022 ± 0.012)	0
Galaxias sp.	8 (0.223 ± 0.065)	8 (0.461 ± 0.098)	8 (0.331 ± 0.095)	11 (0.036 ± 0.017)	12 (0.119 ± 0.070)	8 (0.057 ± 0.030)
H. klunzingeri	0	1 (0.004)	0	8 (0.083 ± 0.195)	11 (0.152 ± 0.243)	6 (0.168 ± 0.277)
H. sp. 'Midgley's'	3 (0.020 ± 0.007)	1 (0.008)	0	0	0	0
M. ambigua	0	0	0	6 (0.018 ± 0.008)	7 (0.020 ± 0.021)	2 (0.037 ± 0.026)
M. anguillicaudatus	0	0	0	8 (0.028 ± 0.016)	12 (0.044 ± 0.044)	5 (0.024 ± 0.003)

M. australasica	0	0	0	0	3 (0.040 ± 0.011)	2 (0.025 ± 0.006)
M. macquariensis	0	0	0	1 (0.015)	2 (0.031 ± 0.039)	1 (0.028)
M. peelii peelii	0	0	0	11 (0.036 ± 0.022)	12 (0.115 ± 0.066)	9 (0.043 ± 0.013)
N. australis	8 (0.183 ± 0.048)	8 (0.231 ± 0.054)	8 (0.183 ± 0.085)	0	0	0
O. mykiss	0	0	0	0	2 (0.055 ± 0.021)	0
P. fluviatilis	8 (0.123 ± 0.068)	8 (0.111 ± 0.058)	8 (0.101 ± 0.029)	2 (0.013 ± 0.000)	3 (0.014 ± 0.004)	1 (0.028)
P. grandiceps	3 (0.017 ± 0.010)	3 (0.013 ± 0.004)	2 (0.044 ± 0.015)	0	0	0
R. semoni	4 (0.018 ± 0.007)	8 (0.069 ± 0.043)	6 (0.061 ± 0.034)	2 (0.010 ± 0.009)	12 (0.161 ± 0.105)	10 (0.074 ± 0.048)
S. trutta	0	0	0	0	3 (0.040 ± 0.017)	1 (0.025)

References

Fuller, S., Baverstock, P., & King, D. (1998). Biogeographic origins of goannas (Varanidae): a molecular perspective. *Molecular Phylogenetics and Evolution*, *9*(2), 294–307. https://doi.org/10.1006/mpev.1997.0476

Hardy, C. M., Adams, M., Jerry, D. R., Court, L. N., Morgan, M. J., & Hartley, D. M. (2011). DNA barcoding to support conservation: species identification, genetic structure and biogeography of fishes in the Murray—Darling River Basin, Australia. *Marine and Freshwater Research*, *62*(8), 887–901.

Jin, X. X., Zhao, S. L., & Wang, R. X. (2013). Universal primers to amplify the complete mitochondrial 12S rRNA gene in marine fish species. *Genetics and Molecular Research*, *12*(4), 4575–4578.

Kearse, M., Moir, R., Wilson, A., Stones-Havas, S., Cheung, M., Sturrock, S., … Drummond, A. (2012). Geneious Basic: An integrated and extendable desktop software platform for the organization and analysis of sequence data. *Bioinformatics*, *28*(12), 1647–1649. https://doi.org/10.1093/bioinformatics/bts199

MacDonald, A. J., Young, M. J., Lintermans, M., & Sarre, S. D. (2014). Primers for detection of Macquarie perch from environmental and trace DNA samples. *Conservation Genetics Resources*, *6*(3), 551–553.

Riaz, T., Shehzad, W., Viari, A., Pompanon, F., Taberlet, P., & Coissac, E. (2011). ecoPrimers: inference of new DNA barcode markers from whole genome sequence analysis. *Nucleic Acids Research*, *39*(21), e145.

Wang, H. Y., Tsai, M. P., Tu, M. C., & Lee, S. C. (2000). Universal primers for amplification of the complete mitochondrial 12S rRNA gene in vertebrates. *Zoological Studies*, *39*(1), 61–66.
